# Audit and Re-Audit of Discharge Summaries Completeness: A Strategy to Improve Patient Care Quality

**DOI:** 10.12669/pjms.41.10.12224

**Published:** 2025-10

**Authors:** Barak Waris, Nauman Ismat Butt, Ayesha Afzal, Imania Khizar

**Affiliations:** 1 Barak Waris, MBBS, House Physician. Department of Medicine & Allied, Chaudhary Muhammad Akram Teaching and Research Hospital, Azra Naheed Medial College, Superior University Lahore, Pakistan; 2 Nauman Ismat Butt, MBBS, CHPE, CHR, FCPS Medicine, FCPS Rheumatology. Assistant Professor, Department of Medicine & Allied, Chaudhary Muhammad Akram Teaching and Research Hospital, Azra Naheed Medial College, Superior University Lahore, Pakistan; 3 Ayesha Afzal, MBBS, House Surgeon. Department of Surgery & Allied, Chaudhary Muhammad Akram Teaching and Research Hospital, Azra Naheed Medial College, Superior University Lahore, Pakistan; 4 Imania Khizar, MBBS, House Surgeon. Department of Surgery & Allied, Chaudhary Muhammad Akram Teaching and Research Hospital, Azra Naheed Medial College, Superior University Lahore, Pakistan

**Keywords:** Audit, Discharge, Patient Care, Re-audit, Surgery

## Abstract

**Objective:**

This study employed an internal audit design to assess the completeness of patient discharge summaries at department of Surgery and allied specialties in Chaudhary Muhammad Akram Teaching and Research Hospital Lahore Pakistan.

**Methods:**

The present audit was conducted at department of Surgery and allied specialties in Chaudhary Muhammad Akram Teaching and Research Hospital, Azra Naheed Medical College, Superior University Lahore Pakistan. Ethical approval was obtained from Institutional Ethical Review Committee maintaining patient confidentiality and data privacy. The initial audit was conducted on 105 discharge summaries of October to November 2024. A standardized checklist was used to assess the completeness the discharge summaries. Following this, targeted interventions aimed at improving documentation practices were done in December 2024. Subsequently, 95 patient discharge summaries from January and February 2025 were included in the re-audit using the same checklist was applied to assess the completeness of discharge summary documentation. Results of both audits were analyzed using SPSS version 23 and compared to determine the level of improvement in documentation completeness.

**Results:**

The initial audit demonstrated compliance in documenting patient’s hospital ID (104, 98.9%), full name (92, 87.6%), contact information (81, 77.1%), admission date (100, 95.2%), discharge date (94, 89.5%). There were gaps in areas such as pending investigations (73, 69.5%) and information on sent biopsies (66, 62.6%). The re-audit demonstrated improvements particularly in pending investigations (84, 88.4%), biopsy information (80, 84.2%) and red-flag symptoms (84, 88.4%), contact information (91, 95.8%) and follow-up appointments (89, 93.7%). However, the presenting complaint (92, 96.8%) and key treatments/procedures (89, 93.7%) showed slight decrease.

**Conclusions:**

The re-audit reflected a positive trend in documentation completeness, with most checklist items showing improved adherence compared to the original audit.

## INTRODUCTION

Good and effective communication is essential for providing quality patient care and hospital discharge summaries play a crucial role in this process. These summaries offer a concise overview of a patient’s hospital stay, highlighting key details like the reason for admission, diagnoses, treatments and post-discharge instructions.^1^ It’s vital that these summaries are both timely and accurate to ensure patient safety and a smooth transition of care once the patient leaves the hospital. A well-crafted discharge summary effectively conveys important information, such as medications, clinical results and follow-up instructions, making it easier for everyone involved.^2^,^3^ On the flip side, poorly written summaries can lead to serious issues, including medication errors, rehospitalization and increased risks of morbidity and mortality.^3^,^4^ The impact of low-quality discharge summaries can be significant, as communication breakdowns are often linked to medical errors, delays in treatment and obstacles to recovery.^4^

It is surprising how often discharge summaries, which play such a vital role in patient care, end up being riddled with errors or missing information all around the globe. In the US, about one-third of these summaries are missing crucial details and in Europe, that number can climb to 40%.^5^,^6^ In Pakistan, the quality of discharge summaries can really vary: some are thorough, while others leave a lot to be desired. A recent study from Pakistan highlighted some common problems, like summaries that are hard to read and lack important information about patients’ other health issues and procedures.^7^ This can seriously put patient safety and ongoing care at risk. One solid way to tackle the issues with discharge summaries is to use a standardized template.^8^,^9^ This approach could help cut down on missing information, reduce human errors and create a more consistent experience across different healthcare environments. Although electronic discharge summaries are starting to make their way into Pakistan, the documentation process still faces hurdles, especially because of inadequate record-keeping and patients not being fully aware of their health conditions, which makes follow-up care even trickier.

Recognizing these clear gaps, we decided to carry out a clinical audit aimed at assessing the quality of our discharge summaries. Clinical audits are incredibly effective for ensuring we meet standards and improve the quality of healthcare services.^9^,^10^ By looking closely at how complete, accurate and timely our discharge summaries are, we aim to identify areas that need improvement and put specific interventions in place. Discharge summaries are crucial for maintaining continuity of care, avoiding medical errors and boosting patient safety. If these documents are inaccurate or incomplete, it can result in rehospitalizations, medication mistakes and a decline in care after discharge. The rationale of our study was to turn our discharge documentation from a potential liability into a vital part of clinical excellence and patient-centered care. We conducted the audit in two cycles, introducing interventions between them to tackle the gaps we found and encourage improvements.

## METHODS

The present study was conducted at department of Surgery and allied specialties in Chaudhary Muhammad Akram Teaching and Research Hospital, Azra Naheed Medical College, Superior University Lahore Pakistan employing an internal audit design to assess the completeness of patient discharge summaries. The audit process was conducted in three stages: an initial audit followed by intervention and subsequently a re-audit to evaluate the improvements in documentation completeness. Medical records with missing patient discharge summaries were excluded from the study.

### Ethics statement:

This study was conducted after ethical approval from Institutional Ethical Review Committee of Azra Naheed Medical College, Superior University Lahore Pakistan prior to data collection. (Ref# ANMC/IRB/2024/034, Dated December 16, 2024) The study followed standards laid down in the 1964 Declaration of Helsinki, revised in the year 2000. Patient confidentiality and data privacy were strictly maintained throughout the study. No patient-identifiable information was used in the analysis and the study adhered to ethical guidelines for healthcare research.

**Fig.1:**
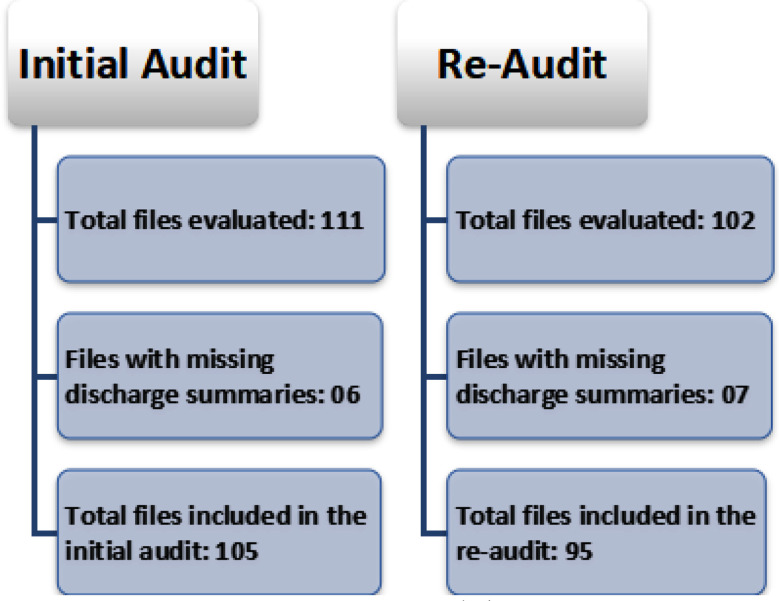
Audit and Re-Audit inclusion process.

The initial audit assessed all medical records for the months of October 2024 to November 2024 using a non-probability consecutive sampling technique. A total of 111 records were obtained, however six records had missing discharge summaries as shown in Fig.1. Subsequently, the initial audit was conducted on a sample of 105 patient discharge summaries. A standardized checklist was used to assess the completeness of various elements of the discharge summaries taking insights from national body of Islamabad Healthcare Regulatory Authority along with international entity of Royal College of Physicians.^11^,^12^ The checklist used to assess documentation completeness included the following 19 items: patient’s full name; patient’s hospital ID; patient’s contact information; date of admission; date of discharge; presenting complaint; admission and discharge diagnoses; key treatments or procedures performed; summary of significant clinical events during hospitalization; significant test results; pending investigations; information on sent biopsies and anticipated results; complete list of discharge medications; doses, frequencies and durations for medications; changes to pre-admission medications; follow-up appointments; red-flag symptoms for the patient to monitor; name of discharging consultant or physician; and signature of the responsible healthcare professional. Each item was marked as ‘documented’ or ‘not documented’ for each discharge summary. The results of this audit were analyzed by calculating the percentage of completeness for each checklist item using SPSS version 23. The percentage was determined by dividing the number of discharge summaries that met the documentation criteria by the total number of records (105) included in the initial audit.

Following the initial audit, targeted interventions aimed at improving documentation practices were done in the month of December 2024. Feedback was provided to the healthcare professionals especially house officers and postgraduate residents involved in documentation and specific training sessions were conducted to address areas of deficiency identified in the initial audit. These interventions aimed to improve documentation practices related to missing or incomplete information in discharge summaries, particularly in areas such as pending investigations, contact information and red-flag symptoms. Subsequently, a re-audit was performed on all medical records for the months of January 2025 and February 2025 using a non-probability consecutive sampling technique. A total of 102 records were obtained, however seven records had missing discharge summaries as depicted in Fig.1. Subsequently, a sample of 95 patient discharge summaries was included in the re-audit. The same checklist was applied to assess the completeness of discharge summary documentation in the re-audit. The results of re-audit were analyzed by calculating the percentage of completeness for each checklist item using SPSS version 23. The percentage was determined by dividing the number of discharge summaries that met the documentation criteria by the total number of records (95) included in the re-audit. The data from both audits were compared to determine the level of improvement in documentation completeness. The percentage change between the audits was then calculated to assess areas of improvement or decline in documentation practices.

## RESULTS

The original audit included 105 discharge files and revealed varying levels of completeness in patient discharge summary documentation as shown in Table-I. Key findings included high compliance in documenting the patient’s hospital ID (104, 98.9%) and the full name (92, 87.6%), though patient contact information was only included in 81 (77.1%) of cases. Other areas such as the date of admission (100, 95.2%), date of discharge (94, 89.5%) and the inclusion of a complete list of discharge medications (101, 96.2%) were well-documented. However, there were gaps in areas such as pending investigations (73, 69.5%) and information on sent biopsies (66, 62.6%).

**Table-I:** Results of audit and re-audit for completeness.

Checklist Questions	Audit Completeness N=105	Re-Audit Completeness N=95	Difference
Is the patient’s full name included?	92 (87.6%)	94 (98.9%)	+11.3%
Is the patient’s hospital ID provided?	104 (98.9%)	95(100.0%)	+1.1%
Is the patient’s contact information included?	81 (77.1%)	91 (95.8%)	+18.7%
Is the date of admission documented?	100 (95.2%)	94 (98.9%)	+3.7%
Is the date of discharge documented?	94 (89.5%)	94 (98.9%)	+9.4%
Is the presenting complaint mentioned?	102 (97.1%)	92 (96.8%)	-0.3%
Are all admission and discharge diagnoses documented?	99 (94.3%)	91 (95.8%)	+1.5%
Are the key treatments or procedures performed during the hospital stay listed?	100 (95.2%)	89 (93.7%)	-1.5%
Are significant clinical events during hospitalization summarized?	89 (84.8%)	89 (93.7%)	+8.9%
Are significant test results included in the summary?	97 (92.4%)	90 (94.7%)	+2.3%
Are pending investigations clearly documented?	73 (69.5%)	84 (88.4%)	+18.9%
Is information on sent biopsy(s) and anticipated results included?	66 (62.6%)	80 (84.2%)	+21.6%
Is a complete list of discharge medications provided?	101 (96.2%)	91 (95.8%)	-0.4%
Are doses, frequencies and durations for each medication included?	97 (92.4%)	93 (97.9%)	+5.5%
Are changes to pre-admission medications clearly documented?	95 (90.5%)	86 (90.5%)	0.0%
Are follow-up appointments specified (date, location)?	93 (88.6%)	89 (93.7%)	+5.1%
Are red-flag symptoms for the patient to watch out for included?	74 (70.5%)	84 (88.4%)	+17.9%
Is the name of the discharging consultant or physician included?	100 (95.2%)	92 (96.8%)	+1.6%
Is the discharge summary signed by the responsible healthcare professional?	93 (88.6%)	92 (96.8%)	+8.2%

The re-audit included 95 discharge files and demonstrated significant improvements across many of these areas, particularly in pending investigations (84, 88.4%), biopsy information (80, 84.2%) and red-flag symptoms (84, 88.4%) as depicted in Table-I. Other notable improvements included patient contact information (91, 95.8%) and follow-up appointments (89, 93.7%). However, the presenting complaint (92, 96.8%) and key treatments or procedures (89, 93.7%) showed slight decrease. Overall, the re-audit reflected a positive trend in documentation completeness, with most checklist items showing improved adherence compared to the original audit.

## DISCUSSION

In the present study, the original audit revealed varying levels of completeness in patient discharge summary documentation showing compliance in documenting the patient’s hospital ID (98.9%), full name (87.6%), while contact information was mentioned in 77.1%. The lack of essential contact details and safety alerts in discharge summaries poses a significant risk to patient safety after discharge. Studies have linked incomplete discharge documentation to higher risks of re-hospitalization, complications and mortality.^7^,^13^ Similar issues of legibility and completeness were also highlighted by La Regina et al.^14^ Other areas such as the date of admission (100, 95.2%), date of discharge (94, 89.5%) and the inclusion of a complete list of discharge medications (101, 96.2%) were well-documented in the present study.

However, there were gaps in areas such as pending investigations (69.5%) and information on sent biopsies (62.6%). The absence of key admission details, including the time and route of admission, reasons for admission and secondary diagnoses raises concerns about their accuracy and usefulness for ongoing patient care. Since primary care providers rely heavily on this information, its omission can result in significant gaps in care, as evidenced by the high rate of readmissions within a short time frame.^7^,^13^ Essential details were omitted in the discharge planning section, such as discharge destination, instructions related to the primary diagnosis and the necessary consultant signatures and counterchecks. These deficiencies emphasize the need for a more organized and dependable discharge planning process. Furthermore, the lack of consistency in scheduling follow-up appointments points to a need for standardized discharge protocols, as the current practices could negatively impact patient follow-up care and health outcomes.^15^,^16^ It should be noted that gaps in in-home medication prescriptions increase the risk of medication errors, which are well-established causes of adverse health outcomes and hospital readmissions.^15^,^17^

In the present study following the original audit, intervention was done to educate the healthcare professionals about the shortcomings. The results of the re-audit demonstrated significant improvements in the completeness of discharge summaries across several key areas. Most notably, there were substantial increases in the inclusion of patient contact information (+18.7%), pending investigations (+18.9%) and biopsy information (+21.6%), reflecting a stronger focus on ensuring comprehensive documentation. Additionally, the completeness of discharge summaries improved in areas such as the inclusion of red-flag symptoms (+17.9%), follow-up appointments (+5.1%) and discharge summary signatures (+8.2%).

While the overall quality improved, some areas showed little to no change, such as documentation of changes to pre-admission medications (0%) and presenting complaints (-0.3%). These results indicate that while substantial progress has been made, further work is needed in ensuring uniformity in documenting all relevant details. Schwarz et al. reported that discharge summaries often lack essential components, especially detailed medication histories and recommendations for further treatment.^16^,^18^ Addressing these gaps, along with eliminating ambiguous abbreviations, is crucial to improving the completeness, clarity and quality of discharge summaries, ultimately enhancing patient safety and continuity of care.^19^,^20^

Another important issue that should be discouraged is the use of ambiguous medical abbreviations. As many as 77% physicians considered medical abbreviations undesirable in discharge summaries, highlighting the need to avoid unclear abbreviations to improve clarity and comprehension.^19^,^20^ The quality of medical documentation especially discharge summaries remains suboptimal in Pakistan, which poses a significant risk to patient safety and continuity of care. Numerous studies have identified key factors attributable to this issue, including time constraints faced by healthcare professionals in limited-resource countries of African and Asia such as Pakistan.^21^,^22^ Other contributing factors encompass inadequate knowledge and lack of formal training in medical documentation during undergraduate and postgraduate medical education and lack of awareness regarding importance of accurate record keeping.^23^,^24^

In addition to this, the prevailing attitude of seeing documentation as a bureaucratic burden rather than a clinical necessity, exacerbates this problem.^25^,^26^ This situation is further compounded by systemic challenges including the absence of standardized templates, limited use of electronic medical records and inadequate institutional policies further compound this situation.^25^,^26^ Improving discharge summary practices requires a multifaceted approach, including structured training programs, incorporation of medical documentation in the medical curriculum, development of user-friendly templates and fostering a culture that values documentation as an essential component of patient care.

### Limitations:

One of the limitations of the present study is its relatively small sample size, which could affect how well the findings apply to other healthcare settings. Moreover, since the audit was carried out in just one institution, the results might differ in various clinical environments. The focus of the audit was solely on completeness, leaving out considerations of the quality or clarity of the information provided.

## CONCLUSIONS

The present clinical audit and re-audit process revealed encouraging improvements in the completeness of discharge summaries, with the most significant gains in areas directly impacting patient safety and follow-up care. However, the persistence of gaps in certain areas highlights the need for ongoing interventions to achieve higher standards of documentation. This process underscores the critical role of discharge summaries in ensuring safe transitions from hospital to home and the importance of continuous quality improvement efforts.

### Recommendations:

Future research could delve into these areas and think about broadening the scope to include a larger and more diverse range of healthcare facilities. Additionally, while some interventions were implemented between the audit cycles, we didn’t quantitatively measure their specific impact, which would be valuable for understanding which strategies were most effective. Based on the findings of our audit, we suggest keeping observing documentation routinely and taking specific actions to tackle the areas that still need some work, like how complaints are presented and changes in medication. It would be beneficial to roll out a stronger training program for healthcare professionals which highlight the need for detailed documentation, which could help bridge these gaps. Plus, fine-tuning the standardized template for discharge summaries could enhance their consistency and completeness, particularly in tricky areas. It is crucial to keep stressing the importance of these summaries for ensuring smooth continuity of care after discharge.

### Authors’ Contribution:

This study was conceived and designed by BW, AA and NIB.

**BW, AA and IK:** Did the initial literature research.

**NIB, BW, AA and IK:** Did the data collection, assembly and medical record assessment.

Data analysis and interpretation were done by NIB, BW and AA.

**BW, NIB, AA and IK:** Were involved in manuscript writing.

**NIB and BW:** Did the final critical review and corrections.

**BW, NIB, AA and IK:** Agree to be responsible and accountable for the accuracy or integrity of the work.
